# Recommendations for occupational therapy interventions for adults with ADHD: a consensus statement from the UK adult ADHD network

**DOI:** 10.1186/s12888-021-03070-z

**Published:** 2021-02-04

**Authors:** Marios Adamou, Philip Asherson, Muhammad Arif, Louise Buckenham, Sally Cubbin, Karina Dancza, Kirstie Gorman, Gísli Gudjonsson, Sharon Gutman, James Kustow, Kerry Mabbott, Teresa May-Benson, Ulrich Muller-Sedgwick, Emma Pell, Mark Pitts, Suzanne Rastrick, Jane Sedgwick, Kath Smith, Clare Taylor, Lucy Thompson, Kobus van Rensburg, Susan Young

**Affiliations:** 1 School of Human & Health Sciences, University of Huddersfield, Huddersfield, UK; 2 MRC Social Genetic and Developmental Psychiatry Research Centre, London, UK; 3 Leicestershire Partnership NHS Trust, Leicestershire, UK; 4 Northamptonshire Healthcare NHS Foundation Trust, Northamptonshire, UK; 5 The Royal College of Occupational Therapy and Heath Care Professionals Council, London, UK; 6 Private Mental Health Clinic, Northampton, UK; 7 Health and Social Sciences Cluster, Singapore Institute of Technology, Singapore, Singapore; 8 Lancashire Care NHS Foundation Trust, Lancashire, UK; 9 Institute of Psychiatry, Psychology and Neuroscience, King’s College London, London, England; 10 Columbia University, New York, USA; 11 Barnet,Enfield and Haringey NHS Trust, London, UK; 12 SPIRAL Foundation, New York, USA; 13 South West Yorkshire Partnership NHS Foundation Trust, Wakefield, UK; 14 South London and Maudsley NHS Foundation Trust, London, UK; 15 NHS England, London, UK; 16 Mind Body Brain Connections, Truro, UK; 17 South West London and St George’s Mental Health NHS Trust, London, UK; 18 Psychology Services Limited, London, UK

**Keywords:** Occupational therapy, Adult ADHD, Multidisciplinary intervention, Sensory intervention, Post diagnostic support

## Abstract

**Background:**

ADHD is neurodevelopmental disorder which persists into adulthood. Presently, therapeutic approaches are mainly pharmacological and psychological whilst the role, scope and approaches of occupational therapists have not been adequately described.

**Results:**

In this consensus statement we propose that by assessing specific aspects of a person’s occupation, occupational therapists can deploy their unique skills in providing specialist interventions for adults with ADHD. We also propose a framework with areas where occupational therapists can focus their assessments and give practice examples of specific interventions.

**Conclusions:**

Occupational therapists have much to offer in providing interventions for adults with ADHD. A unified and flexible approach when working with adults with ADHD is most appropriate and further research on occupational therapy interventions is needed.

## Background

Attention-deficit/hyperactivity disorder (ADHD) is a common neuropsychiatric disorder with a pooled worldwide prevalence estimated at approximately 5% in school-aged children. The symptoms of childhood ADHD are found to persist in adulthood in up to 65% of cases, leading to a prevalence of the condition in that population to be approximately 2.5% [1].

Attention-deficit hyperactivity disorder (ADHD) is characterised by clinical impairment in the areas of inattention and/or hyperactivity–impulsivity [2] and is associated with deficits in executive function, emotional regulation, and motivation [3].

Many adults with ADHD and are used to their lifelong symptoms, have a limited awareness of how ADHD adversely affects their life; some report higher symptoms but lower impairments or vice versa and this may affect diagnostic accuracy. Also, adult diagnoses may be missed in clinical practice due to lack of knowledge about ADHD in adulthood among practitioners and due to the high frequency of comorbid psychiatric conditions [4].

If an adult receives a diagnosis of ADHD, the treatment options open to them which are supported with robust evidence base is either or a combination of pharmacological or psychological [5]. Despite evidence base suggesting that occupational therapy is beneficial if applied as an approach in other mental health disorders, not much exists for adults with ADHD. We think however that such interventions are not only requested by service users, but also have a robust clinical basis to make them essential in providing interventions to adults with ADHD.

## Method

The consensus aimed to provide practical guidance to occupational therapy professionals working with adults with ADHD, drawing on the scientific literature and the professional experience of the authors. To achieve this aim, professionals specialising in ADHD convened in London at expert workshop called “Occupational Therapy and Adult ADHD” on the 10th February 2017. The event was hosted by the UK Adult ADHD Network (UKAAN). UKAAN is an organisation founded in 2009 by a group of mental health specialists in response both to the NICE guidelines [6] (now amended [5]) and to recommendations from the British Association for Psychopharmacology (BAP) [7] (now amended [8]) that aims to provide support, education, research and training for mental health professionals working with adults with ADHD.

Meeting attendees included experts in ADHD across a range of mental health professions, including healthcare specialists (nursing and adult psychiatry; clinical and forensic psychology; counselling), academic, educational and occupational specialists. Attendees engaged in discussions throughout the day, with the aim of reaching consensus.

The day was structured around presentations on pre-selected topics of interest by invited experts, followed by a discussion after each presentation aiming to reach a consensus position on the topic. At the end of the day, there was a summary presentation of the points previously agreed and further discussion.

The meeting started with a review of the status of non-pharmacological interventions in adult ADHD to set the scene. Then experts presented the following topics:

1. Recovery approaches in adult ADHD.
2. AHP into action: A Strategic Framework and Opportunity
3. Occupational Therapists as Healthcare Professionals: roles, expertise, skills, future developments in the professions.
4. Adult ADHD: implications for O/T interventions
5. Praxis difficulties in adult ADHD
6. Sensory integration difficulties in adult ADHD

The consensus group incorporated evidence from a broad range of sources. All consensus proceedings were audio-recorded and transcribed.

## Results and consensus outcome

### Occupational therapy in the wider healthcare system in the NHS - allied health professionals

Allied Health Professionals (AHPs) are health care professionals distinct from nursing, medicine, and pharmacy [9]. They are the third largest workforce in the NHS. In the main they are degree level professions and are professionally autonomous practitioners. Presently, 13 of the 14 AHPs are regulated by the Health and Care Professions Council (HCPC) with Osteopaths regulated by the General Osteopathic Council (GOC). Among other roles they are involved with the delivery of health or related services pertaining to the identification, evaluation and prevention of diseases and disorders, dietary and nutrition services, rehabilitation and health systems management.

A recent strategy developed to inform and inspire the healthcare system about how AHPs can be best utilised to support key healthcare transformation initiatives [10] suggest they can be impactful by 1. improving the health and wellbeing of people and populations 2. supporting and providing solutions to general practice and urgent and emergency services to address demand 3. supporting integration, addressing historical service boundaries to reduce duplication and fragmentation 4. delivering evidence based practice to address unexplained variances in service quality and efficiency.

To deliver this work, the AHPs have entered into four commitments (to the individual, to keep care closer to home, to the health and wellbeing of population and to care for those who care) and four priorities (to lead change, further develop their skills, utilise information and technology and evaluate, improve and evidence the impact of their contribution). Occupational Therapists are essential members of AHP group and committed to support the strategic objectives and priorities so their impact and contribution in the wider healthcare system is enhanced.

### Occupational therapy practice and adult ADHD

The ability to synthesise and apply occupational concepts is what uniquely distinguishes occupational therapy from other health professions [11, 12]. The primary goal of occupational therapy is to enable people to participate in the activities of everyday life. Occupational therapists achieve this outcome by working with people and communities to enhance their ability to engage in the occupations they want to, need to, or are expected to do, or by modifying the occupation or the environment to better support their occupational engagement [13]. There now a renewed understanding of how engagement in occupation is therapy and fundamental to health and wellbeing [14].

Occupational therapy practice emphasises the occupational nature of humans and the importance of occupational identity [15]. It provides practical support to empower people to facilitate recovery and overcome barriers preventing them from doing the activities (or occupations) that matter to them and also utilises occupation to maintain health or prevent deterioration.

This support increases people’s independence and satisfaction in all aspects of life. “Occupation” as a term refers to practical and purposeful activities that allow people to live independently and have a sense of identity [16, 17]. This could be essential day-to-day tasks such as self-care, work or leisure.

Occupations are central to a person’s identity and sense of competence and have particular meaning and value to that individual. Occupational therapists are skilled in evaluating all aspects of the domain, their interrelationships, and the client within his or her contexts and environments (Table 1). Originally founded on humanistic values, occupational therapy emphasised occupation as the positive engagement between the person and the environment to influence overall well-being [11, 18] whilst other definitions followed and can add to an understanding of this core concept [14, 19, 20]. Occupations occur in context and are influenced by the interplay among factors of the individual, performance skills, and performance patterns. Occupations occur over time; have purpose, meaning, and perceived utility to the client; and can be observed by others (e.g., preparing a meal) or be known only to the person involved (e.g., learning through reading a textbook). Occupations can involve the execution of multiple activities for completion and can result in various outcomes.

**Table 1:** Aspects of the domain of occupational therapy. All aspects of the domain transact to support engagement, participation and health

Occupations	Client Factors	Performance Skills	Performance Patterns	Contexts and Environments
Activities of daily living (ADLs)^a^ Instrumental activities of daily living (IADLs): Rest and sleep, Education, Work, Play, Leisure, Social participation	Values, beliefs and spirituality, Body functions, Body structures	Motor skills, Process skills, Social interaction skills	Habits, Routines, Rituals Roles	Cultural, Personal, Physical, Social Temporal, Virtual

^a^Also referred to as basic activities of daily living (BADLs) or personal activities of daily living (PADLs)

ADHD affects all aspects of Occupational Functioning: In terms of Client Factors, people with ADHD report low self-esteem [21] and self-efficacy [22]. In terms of Contexts and Environments, it affects *educational* functioning [23] with studies in childhood demonstrating disruptive classroom behaviour and academic underperformance, poor grades, poor reading [24] and overall, adverse long-term effect on academic outcomes [24–26]. Similarly ADHD affects *relationships* [27] and there is evidence that these are particularly affected in the ability to provide emotional support and manage interpersonal conflict [28] which can lead to divorce [29] and loneliness [30].

In terms of Performance Patterns, referring to *employment*, there is evidence to imply poor performance; for example, young adults with ADHD were shown to change employment frequently, obtain fewer full time occupations and be more frequently fired [31]. Similarly, in a follow up study of boys with ADHD aged 4–12 who were initially treated at a university medical clinic, 41% had been fired at least once and 26% were unemployed at follow-up during ages 21–23 [32].

Another study estimated that adult ADHD was associated with a 4–5% reduction in work performance, a 2.1 relative-odds of sickness absence and a 2.0 relative-odds of workplace accidents and injuries [33]. A survey undertaken by the World Health Organisation in 10 countries reported that 3.5% of the workers suffered from ADHD resulting in 143 million days of lost production. Workers with ADHD had an average 8.4 excess sickness absence days per year and even higher annualised average excess numbers of work days associated with reduced work quantity (21.7 days) and quality (13.6 days). In addition to this, ADHD has been associated with increased absenteeism [34, 35], impaired organisational skills [36] and abilities [37] and poor time management [38].

A possible explanation why these Contexts and Environments are affected, may be due to the cognitive impairment which has been documented in ADHD which leads to Performance Skills impairment. We know that both vigilance and sustained attention are impaired in adults with ADHD [39] so it is expected that ADHD will interfere with task performance due to attentional deficits. Also, impairment is found in cognitive flexibility or set shifting referring to the ability to switch attention from one aspect of an object to another, or to adapt and shift one’s response based on situational demands, such as changes in the rules, schedule, or type of reinforcement in a task [40, 41].

One of the well documented deficits in ADHD is to their executive function; this is what allows an individual to plan a series of steps necessary to achieve a desired goal, keep these steps in mind whilst acting on the goal, monitor progress through these steps, and have the cognitive flexibility to adjust or change the steps if progress is not being made toward the original goal [42]. In adults with ADHD, deficits to these functions have been well studied [43, 44].

Apart from deficits in attention and executive function, children [45] and adults [46] with ADHD also have working memory deficits which can affect performance.

Occupational therapists have skills and competencies unique in understanding and affecting change in adult ADHD which affects many domains of a person’s occupation. Although the theoretical case can be made, there is need for further research to evidence the effects for occupational therapy interventions in adult ADHD.

### Occupational therapy practice models and adult ADHD

Best practice requires that therapists thoughtfully choose the models that fit their views of the purpose and focus of therapy, as well as support their ability to understand and explain the specific challenges faced by their clients [47]. “The therapist should collaborate with the person to establish the priority occupational areas which will be the focus of occupational therapy intervention. Assessment of current performance in these occupational areas will guide appropriate intervention strategies, which may focus on compensating for the challenges, developing skills or enhancing/developing performance components.” Subsequently, the occupational therapist may draw from a range of suitable models to guide intervention Occupational therapy has a wide variety of models available to understand the people’s occupations. There is no consensus as to the single model which should be used in all circumstances. The Canadian Model of Occupational Performance (CMOP) [48], Person-Environment-Occupation-Performance Model (PEOP) [49, 50] and Model of Human Occupation (MOHO) [51] models are all acceptable in practice although a unified and flexible approach is recommended for people with Adult ADHD. A therapist who begins with an occupation-focused model as the organising model of practice will have gathered essential information about occupational roles and priorities up front and will be reminded to ensure that therapy sessions reflect client-centred goals and interests. This client-centred therapy focus fits well with the recent emphasis on patient-centred measures of satisfaction in healthcare [52].

### Occupational therapy approaches and adult ADHD

Interventions for ADHD are mainly focused on how symptom reduction can be achieved with either Medicines or Psychological Interventions [5]. However although useful, these do not provide guidance on how interventions can be structured to deliver ‘real life’ benefits beyond symptom reduction or increase participation, bearing in mind that symptom reduction alone does not always produce improvement in daily functioning [53].

From previous work, we found that the framework proposed by the ADHD Star can be a useful guide to multidisciplinary interventions based on the ADHD Star domains ‘Focus and Attention’, ‘Friends and Social Life’, ‘Physical Health’, ‘How you Feel’ “Understanding your ADHD”, “Organising yourself”, “Thinking and reacting” and “Meaningful use of time” [54]. To this framework, the Occupational Therapy Models of Practice can be deployed using the different frames of reference depending on the needs of the individual.

*Interventions* consider what the therapist and client identify to work on during a treatment session. Interventions are defined from the Occupational Therapy Practice Framework [55] and include preparatory methods, purposeful activities and occupation-based interventions. *Preparatory methods* are techniques that prepare a client to participate in occupations and for our purpose can be a discussion and completion of the ADHD Star. *Purposeful activities* suggest that the client participate in activities that help improve skills that would enhance occupational performance, such as gardening, joining a social group, doing voluntary work etc. *Occupation-based intervention* is when a client, in therapy, engages in occupations that match his or her identified goals, which may include cooking in a kitchen, getting dressed in his or her room, and travelling independently. Observations are made on how the therapist describes the interventions to the client and if they relate to the client’s goals. The Occupational therapist will use a graded approach and adapt the demands of the occupation, the environmental context or the support provided to maximise independence, skill acquisition and self-efficacy.

## Discussion

In considering which intervention approaches to recommend for ADHD, we used our clinical experience and the strategies suggested by Fleming [56] to solve therapy issues and decide how the intervention session should flow: procedural, interactive and conditional reasoning. We also noted that clients can be dissatisfied with traditional therapy that is not grounded in meaningful occupations [57]. In healthcare settings, occupational therapists collaborate with many other professionals to help individuals on their road to recovery. While the role of the occupational therapist may overlap with other team members, the occupational therapist provides a unique theoretical and clinical contribution to the recovery and treatment team; thus, occupational therapy should be considered a vital part of a comprehensive and integrated treatment program. Interface with other professionals can take place at multidisciplinary team meetings and can help delinate who is best suited to provide interventions.

We propose that occupational therapy for adults with ADHD should focus on the following:

1. Organising and adapting the physical and /or social environment to enable participation 2. Promoting social awareness and interactions within occupations 3. Encouraging self-management of symptoms through strategies such as sensory regulation, stress management techniques, adaptations to routines.

### Organising their environments

The fact that treatment environments influence a client and their performance has been known for many years [58]. In the context of this paper, environment is defined as the “particular physical and social features of the specific context in one does something that impacts upon what one does, and how it is done” [59]. Occupational therapists can use several models to describe interactions between the person, occupation and environment, which can be applied to a practice situation [60]. As most people operate in a variety of environments in the course of the day (physical, social, cultural), interventions should consider assessments and interventions in more than one.

For example, a common phenomenon of ADHD is an inability to organise one’s physical environment to support daily functioning. Adults with ADHD frequently report that their physical environment such as their workplace, homes, car interior and personal belongings (e.g., wallets, handbags, bookbags) are very untidy and that despite their efforts to organise or maintain their physical environments over prolonged period, their attempts are not successful. Occupational Therapist led interventions can help individuals understand that organisation requires regular, repeated behaviours rather than just one singular attempt. Creating structure, establishing routines and assigning places for essential items are often key to maintaining organisation. Occupational Therapists can use their unique skills to help establish behavioural change to support organisation, for example: having regular set times to sleep, eat and leave for work.

Not having set times for these activities can lead to an unstructured and disorganised day. Occupational therapists can also introduce colour-coded label systems to allow for easy identification and location of items, and work with the individual to create file and record systems to store important documentation, such as utility bills or medical appointments. This reduces the clutter from a physical space, as well as allowing for easy access to these documents.

### Enhance social interaction and awareness

Social interactions of people with ADHD are often impaired. For example children with ADHD are reported to have problems with peer relations [61], are less well liked [62], have mood dysregulation [63] emotional lability [64, 65], friends report less positive friendship quality [66], and have higher oppositional defiant scores [67] whilst adults may have fear of intimacy [68], general impairment in social functioning [69–72] and some even suggested ADHD may be an early stage of a personality disorder [73].

During social interaction, the person with ADHD already operates within a sense of personal capacity (self-assessment of one’s physical, intellectual and social abilities [74] and self-efficacy (the thoughts and feelings one has concerning perceived effectiveness in using personal abilities to achieve desired outcomes) [75] which is impaired although the level of the impairment or its awareness may be variable according to the individual.

Interventions in this domain of should promote self-efficacy and increased appraisal of the self. Self-efficacy relates to how one uses capacity to impact on what happens in life and depends on the individual’s sense of self control and impact of efforts. Due to lack of insights into their deficits, there is often resistance and denial from the individual during this process, requiring the use of the professional’s therapeutic skills “To increase self-efficacy, occupational therapist support the person to do their needed occupations such as paying bills, planning meals or meeting job requirements.”

In terms of personal capacity, tasks that promote clear roles in controlled social conditions such as voluntary work or specifically commissioned community projects can provide opportunities for clearly defined activity choices which can establish new patterns of action which may then crystallise into occupational choices. This can lead the person with ADHD to enter a new occupational role or acquire a new habit. Often role-play can be used to demonstrate and practise the norms of social interactions for specific situations, until enhanced personal capacity is achieved.

In addition, Occupational Therapists can also provide education to family members about the individuals’ experiences and provide reasons for their behaviours; often these can be mistaken for passive aggressive or avoidant behaviours, or as an intentional act against someone.

### Develop stress management techniques

In the context of Occupational Therapy, these techniques relate *to planning the day* and *time management*.

It has already been identified that the psychological aspects of the stress response in adults suffering from ADHD are impaired [76] with individuals having high self-perceived stress [77] and many stressors [78]. Stressful situations can lead to heightened disorganisation, disinhibition, inattention and mood liability, which can exacerbate ADHD symptoms.

#### Planning the day

One of the most important strategies for adults with ADHD to reduce stress it to operate in a planned mode rather than a reactive mode. A planned mode is characterised by (a) developing a planned schedule for each day, (b) adhering to the schedule being mindful that distractions can affect one’s plans, and (c) understanding when it might be appropriate to exercise flexibility once a schedule is decided. In a reactive mode, people would allow distractions to alter their adherence to a schedule, take up activities based on which one arises last without completing any and just let someone else to organise their daily schedule. In this reactive mode, people just live managing daily crises. Learning to structure their life in a planned mode, rather than impulsively reacting to events as they arise is a critical skill for adults with ADHD along with the ability to remain calm and stop and think before reacting. It may take time to help adults with ADHD learn that every situation does not require immediate attention or response, and that prioritising daily demands can be a useful stress management skill.

To achieve a planned mode, adults with ADHD need to develop new familiar and automatic aspects of daily life through a process of habituation. Habituation is defined as an internalised readiness to exhibit consistent patterns of behaviour guided by our habits and comes about as a consequence of repeated patterns of behaviour under certain temporal, physical and sociocultural contexts [79] Occupational therapists can help clients consistently plan recreation/relaxation time into their schedules even if such planned periods consist of only 15 min to release restless energy and frustration in appropriate ways rather than transferring these emotions to relationships with others. The use of relaxation techniques (e.g., deep breathing exercises, meditation, engagement in sports) is frequently helpful to clients experiencing some degree of hyperactivity or restlessness. Daily engagement in relaxation/recreation also promotes a greater lifestyle balance-an ingredient of stress management that is frequently missing in the lives of adults with ADHD.

#### Optimise time management

ADHD has been associated with poor time perception [80–82] and it has been noticed that children with ADHD have difficulties in developing a schedule to complete tasks in a timely fashion, remembering tasks and materials, and setting priorities [83]. It has also been suggested that stimulant medication may bring some benefits in time management [36] in children but overall, other interventions may be more beneficial. For example, Occupational Therapists can work with individuals to address effective time management skills and develop discipline to adhere to planned activities despite more appealing distractions. This can be achieved through helping the individual to understand and practise the skills of time management, involving planning a schedule for responsibilities, self-care, and exercising flexibility to change these plans. Typically developing individuals are generally able to take a more pre-emptive approach and are able to complete their set tasks on time and can more accurately predict time and task completion time but this can prove difficult for adults with ADHD. Often, they can become so absorbed in one activity, that they’re unaware of time elapsing, which impacts on their daily schedule. This dysfunctional time management can often contribute to chronic lateness in adults with ADHD, which can impact on their employment or attendance important appointments. Over commitment could contribute to chronic lateness, as does distractibility. Distractibility is another contributor to chronic lateness often observed in adults with ADHD, as non-essential activities can take precedence over planned ones. Occupational Therapists can offer their skills to teach clients how to use electronic alert systems, for example, to indicate when a task should end. Occupational Therapists can also use their therapeutic skills to help individuals to better understand their reasons for avoiding certain activities, and to practise the skills needed to reduce these behaviours. For example, an individual may avoid initiating a task as they find it “mentally draining”. Occupational Therapists can teach individuals to break-down activities into small, manageable tasks, in order to enhance their use of time and reduce sensory overload, or to introduce scheduled and regular breaks.

### Monitor and regulate sensory stimulation

Sensory Integration theory links neurological processes to functional behaviour, whereby sensations from within an individual’s body and their environment are processed to enable them to participate in everyday life. Successful Sensory Integration is an adaptive process and follows when information from several of the senses travels into the brain to be processed. There is evidence that children with ADHD have sensory sensory processing problems [84] and the same seems to continue to adulthood [85] which may require specialist intervention.

Occupational therapists can help adults with ADHD to recognise the relationship between mood, performance, and sensory stimulation; and learn to monitor and regulate the amount and type of sensory stimulation that enters their nervous system at any one time. The latter skill involves the concept of a sensory diet–or the ideas that one’s day is comprised of many different types of sensory experiences that can have a cumulative effect, and that people have unique sensory requirements that must be met to function optimally in all occupational domains [86]. For example, if adults with ADHD have hypereactivity to sensory stimulation, this can directly influence an individual’s mood, alertness and performance level – often without their awareness or understanding. Sensory over load can often result in emotional eruptions, impulsive decision-making and poor concentration, and occurs when an individual struggle to process the volume of sensory information from their environment. Sensory hyporeactivity occurs when individuals have not been exposed to sufficient sensory stimulation to maintain alertness and optimal performance; this can manifest as boredom, restlessness and inattention. Occupational Therapists can work together with individuals to recognise the common situations in which sensory hyper or hypo reactivity can occur, and the steps they can take to ameliorate the effects on their ADHD symptoms. A key role of the Occupational Therapist is to promote self-regulation and support clients to recognise when they are in a calm alert state, moving into over-alert or under-alert state and what activities and behaviours support self-regulation back to a calm alert state. For example, taking regular breaks to allow for physical movement that provides proprioceptive input can help shift from both over-alert and under-alert into a calm alert state and optimum performance. Occupational Therapists can also offer advice for the workplace, in terms of reasonable adjustments and/or modifications to the environment to reduce sensory input. A simple strategy of headphones or screen panels can reduce auditory and visual input and reduce distractions.

## Conclusions

Occupational therapists have much to offer in providing interventions for adults with ADHD. They are part of a unique group of professionals outside medicine, nursing and psychology with much to offer in delivering care. Occupational therapists have a unique undersdanding of the human as an occupational being and are able to rely on established models of practice. Currently, the evidence base for adults with ADHD is still being developed and this consensus statement aims to bridge that gap by providing practical recommendations for the jobbing occupational therapy on how to approach ‘what works’ for this service user group.

What we propose is a unified and flexible approach when working with adults with ADHD. It is already known that for adults with ADHD to engage with activities these need to be perceived as inspiring, include an apparent goal to stimulate performance and need facilitating support [87]. What our work suggests adds it the areas where this work should focus. This might include work relating to organising their environments, enhancing social interaction/awareness, developing stress management techniques and monitoring/regulating sensory integration for the individual.

We also identified a need for occupational therapists to conduct research into the proposed consensus-based areas of intervention to better demonstrate the efficacy of their approach.

## Data Availability

Data sharing is not applicable to this article as no data set were generated or analysed during the study.

## Abbreviations

ADL: Activities of daily living
ADHD: Attention-deficit/hyperactivity disorder
AHP: Allied Health Professional(s)
BAP: British Association for Psychopharmacology
BALDs: basic activities of daily living
CMOP: Canadian Model of Occupational Performance
GOC: General Osteopathic Council
HCPC: Health and Care Professions Council
IADLs: Instrumental activities of daily living
MOHO: Model of Human Occupation
NHS: National Health Service
NICE: National Institute for Health and Care Excellence
PADLs: personal activities of daily living
PEOP: Person Environment Occupation Performance Model
UKAAN: UK Adult ADHD Network
